# Associations of Sleep with Food Cravings, Diet, and Obesity in Adolescence

**DOI:** 10.3390/nu11122899

**Published:** 2019-11-30

**Authors:** Chelsea L. Kracht, Jean-Philippe Chaput, Corby K. Martin, Catherine M. Champagne, Peter T. Katzmarzyk, Amanda E. Staiano

**Affiliations:** 1 Pennington Biomedical Research Center, 6400 Perkins Road, Baton Rouge, LA 70808, USA; chelsea.kracht@pbrc.edu (C.L.K.); corby.martin@pbrc.edu (C.K.M.); Catherine.Champagne@pbrc.edu (C.M.C.); peter.katzmarzyk@pbrc.edu (P.T.K.); 2 Healthy Active Living and Obesity Research Group, Children’s Hospital of Eastern Ontario Research Institute, 401 Smyth Rd, Ottawa, ON K1H 5B2, Canada; jpchaput@cheo.on.ca

**Keywords:** adiposity, healthy eating index, appetite, child

## Abstract

*Background*: Sleep and dietary intake/quality can contribute to excess weight gain, but food cravings may influence these relationships. This cross-sectional study examined the relationship of adolescents’ sleep characteristics with dietary intake/quality and obesity and whether food cravings mediated these relationships. *Methods*: Sleep measures were calculated based on 24-h accelerometry, and height and weight were directly measured to calculate body mass index (BMI) z-scores. Food cravings were assessed by the Food Craving Inventory (FCI). Dietary intake and quality were calculated based on dietary recalls. Multivariable linear regression was used to examine the associations among sleep, food cravings, dietary intake/quality, and obesity, adjusting for confounders. *Results:* In total, 256 adolescents (ages 10–16 years) had complete data; 42% were non-White and 45% were boys. Sleep efficiency was inversely associated with sweet cravings and FCI-28. Sleep duration, meeting the sleep duration guidelines, and fruit/vegetable cravings were each positively associated with dietary quality. Sleep duration was negatively associated with BMI z-score. Mediation models were not performed as no sleep parameter was associated with both cravings and dietary intake/quality or BMI z-score. *Conclusions:* Associations existed among poor sleep, quantity and quality, with more frequent food cravings and worse dietary quality. Sleep may underlie adolescent obesogenic behaviors.

## 1. Introduction

Adolescence is an important time in development but is often characterized by unhealthy, obesogenic behaviors. Consuming a healthy diet is imperative at this age, as dietary intake influences cardiometabolic risk factors [1] and excess weight gain [2], setting the stage for future health. Despite the importance of this time period for healthy growth, adolescents tend to have poor quality diets and few meet the U.S. dietary guidelines [3]. In addition to poor diets, many adolescents are also getting insufficient sleep. The majority of U.S. adolescents (68.8%) ages 14 to 18 years sleep less than the national sleep guidelines [4,5]. Aside from hours of sleep, the pattern of sleep may also be important, including the quality of their sleep, time of day that an adolescent falls asleep or awakens, and the consistency in total hours across days, all of which have been shown to be associated with obesity in adolescents [6]. The goal of the present study was to examine the inter-relations among sleep, diet, and obesity in adolescents.

Sleep and dietary intake/quality may be inter-related, as quality and timing of dietary intake at night may delay or shorten sleep duration [7]. In studies of adolescents, this delayed sleep may influence breakfast timing and quality [8], and skipping breakfast is associated with lower dietary quality in adolescents [9]. A previous clinical trial in youth ages 8 to 11 years targeting improvements in sleep resulted in healthier dietary intake [10], whereas when sleep was restricted in adolescents ages 14 to 16 years these youth exhibited poorer dietary intake [11]. Aside from logistical considerations of sleep and dietary intake/quality, there is another potential intermediary linking sleep and diet intake/quality that has yet to be tested. This intermediary is food cravings, which are an intense desire to consume a particular food item and have been demonstrated as an important predictor for weight gain in adults [12]. Cravings may be impacted by sleep and, in turn, influence dietary intake and dietary quality. In a recent experimental study of adults, less sleep was related to taste and wanting for high fat and sweet foods [13]. Age is an important factor when considering cravings, as cross-sectional evidence in young adolescents and adults (ages 6 to 23 years) found that with increasing age there are less frequent food cravings [14]. Consequently, recent literature has suggested a focus on understanding cravings in adolescents [15], as cravings may be more frequent and thus more influential on food intake compared to associations observed in adults. Investigations to date into the relationship between sleep and food cravings in adolescence have focused on sleep amount during day and night hours [16], rather than delay of sleep or other sleep parameters which may be also contribute to dietary intake/quality.

As it relates to food cravings and dietary quality or obesity, most published studies are behavioral interventions that evaluate cravings as a potential intermediary for brain processes related to dietary quality and/or reducing energy intake among adults [17] or specifically adults with obesity [18]. There is limited evidence of specific healthy or unhealthy cravings being directly linked to dietary quality in any age group. Most studies are conducted in adults or under the conditions of weight loss, while the relationships between multiple sleep parameters, cravings, and dietary quality in adolescents are still untested. Further, prior data on sleep duration and sleep patterns during adolescence were primarily measured via self-report [6,16,19], and objective measures of sleep are needed. Therefore, the purpose of this paper was to examine potential associations among accelerometry-measured sleep with food cravings, dietary intake/quality, and obesity in adolescents. We hypothesized that food cravings would mediate the relationship between sleep, dietary intake/quality, and body mass index (BMI) z-scores.

## 2. Methods

### 2.1. Participants

Adolescents 10–16 years of age were recruited and enrolled between August 2016 and August 2018 from a city in a southeastern U.S. state to participate in a prospective observational cohort study. Baseline measures from this cohort were used for the present cross-sectional analysis as follow up data collection is ongoing. Parents of potentially eligible adolescents were recruited via several convenience sampling strategies including email, health fairs, and social media. Exclusion criteria included a body weight of more than 227 kg, pregnancy, on a medically restrictive diet, and significant physical or mental disabilities that may impede walking or wearing an accelerometer. The study was approved by the Pennington Biomedical Research Center’s Institutional Review Board.

### 2.2. Procedures

After the parent/guardian and participant provided written informed consent and assent, respectively, the adolescent was instructed to wear an a GT3X+ accelerometer (ActiGraph, Pensacola, FL, U.S.A.) on their hip for seven continuous days (24 h per day) and complete online dietary recalls for 3 days. After seven days, the adolescent and the parent attended a baseline clinic visit, which included anthropometric measurements and questionnaires. Parents completed a questionnaire regarding demographics, including the adolescent’s age, sex, race, and the annual household income. Adolescents reported whether they were in or out of school at the time of measurements. Study questionnaires were administered and managed using Research Electronic Data Capture (REDCap), an electronic data capture tool hosted at Pennington Biomedical Research Center. REDCap is a secure, web-based application designed to support data capture for research studies [20]. Height and weight measurements were completed by a trained researcher and were measured to the nearest 0.1 cm or 0.1 kg, respectively. Age- and sex- adjusted BMI percentiles and BMI z-scores were calculated based on national reference data [21]. Obesity was defined as having a BMI percentile that was greater than or equal to the 95th percentile and overweight was defined as a BMI percentile greater than or equal to the 85th percentile [21].

### 2.3. Sleep Behavior and Physical Activity

Adolescent accelerometry data were evaluated using a validated algorithm to classify sleep and non-wear time in youth [22,23]. Adolescents with at least 3 days (including 1 weekend day) of 160 sleep minutes based on the validation of the algorithm were included in analysis. The algorithm was validated within a younger adolescent sample (9–11 years of age). One hundred and sixty minutes per night was designated as the minimum for inclusion as this would be the minimum amount of time needed to detect a single sleep phase [23]. The algorithm was conducted by a trained statistician using minute-level sleep data and SAS 9.4 software (Cary, NC, U.S.A.).

To adequately capture physical activity, adolescents with at least 4 days (including 1 weekend day) of 10 h per day of wear time (not including sleep or non-wear time) were included in the analyses based on previous studies [24]. For physical activity, fifteen second epochs of accelerometry data not spent in non-wear or sleep were analyzed using age-appropriate cut points to determine movement intensity, including moderate-to-vigorous physical activity (MVPA), which was determined as greater than 574 counts/15 s [25]. Average MVPA (minutes) per day was used as a covariate in adjusted models.

#### 2.3.1. Sleep Duration and Variability

Sleep duration was calculated as the total sleep episode time, defined as total time spent between estimated bedtimes and wake times based on accelerometry between a 12:00 p.m. to 12:00 p.m. period averaged over the number of complete days. The National Sleep Foundation guidelines are 9–11 h for adolescents below 14 years of age and 8–10 h for adolescents 14 years or older [5]. Adolescent sleep was classified as meeting these guidelines across all available days. Sleep variability was assessed as the difference between weekday and weekend sleep hours by calculating the difference between average weekday sleep duration (hours) and weekend sleep duration (hours). A positive sleep variability amount indicates more hours slept on the weekend compared to the weekday (also referred as catch-up sleep), whereas negative sleep variability indicates fewer hours slept on the weekend compared to weekdays.

#### 2.3.2. Sleep Efficiency and Late Bedtime

Sleep efficiency was calculated as the proportion of total sleep episodes (continuous intervals of sleep) divided by sleep period time (expressed in %) and was determined via the validated algorithm [22,26]. A higher sleep efficiency indicates that more of the sleep period was spent in a sleep episode. To compare late bedtimes, adolescents whose average bedtime was later than 10:00 p.m. were classified as “late bedtime” to allow at least 8 h of sleep before a typical school start time (7:00–7:30 am) in the local high schools of the predominant city of the participants, similar to previous studies [27]. Adolescents whose average bedtime was before 10:00 p.m. were classified as “regular bedtime”.

### 2.4. Food Cravings and Dietary Quality

Adolescents completed the Food Craving Inventory (FCI) [28], which assesses the frequency of cravings for specific types of foods over the past month. This survey was completed as part of the clinic visit that participants completed in a fasted state. Responses on the FCI are via a 5 point scale from 1 *= never* and 5 = *always/almost every day*. The questionnaire contains five subscales that quantify cravings for foods that fall into the following categories: high fat foods (8 foods), sweets (8 foods), carbohydrates/starches (8 foods), fast foods (4 foods), and fruit and vegetables (5 foods). This tool has been previously validated in a predominantly late adolescence and emerging adulthood sample (average age 20–30 years) [28]. Subscale scores and a total score (FCI-33) reflecting general cravings were calculated by taking the average ratings for foods in the various subscales, with a higher score indicating that the cravings occurred more frequently. The high fat, sweets, carbohydrates/starches, and fast foods question responses were averaged to create a total score that did not include fruit/vegetable cravings (FCI-28).

Adolescents were asked to complete two days of food and beverage recalls at home and one dietary recall at the research center using the Automated Self-Administered 24-Hour Dietary Assessment Tool (ASA-24 2016), which is a web-based system developed by the National Cancer Institute to administer 24-h dietary recalls [29]. Ahead of the baseline clinic visit, dietary recalls were sent via email at prescheduled intervals with the focus for adolescents to complete one weekday and one weekend day before the visit (which occurred on a weekday). The ASA-24 has been previously validated in this age range and uses the Automated Multiple Pass Method, which uses multiple prompts to elicit recall of food and beverages consumed throughout the prior day [30]. Healthy Eating Index (HEI) 2015 was calculated using food equivalent information provided by ASA-24 and a published SAS Macro [31]. HEI total score is a calorie-adjusted measure based upon the Dietary Guidelines for Americans in 2015, consisting of 13 subcomponents that create a total score of 0 to 100 with a higher score indicating healthier dietary quality. Kilocalories or “energy intake” per day was averaged across all days that the adolescent completed the dietary recalls.

### 2.5. Statistical Methods

Descriptive characteristics were assessed using central tendencies and frequency measures. Pearson correlations were computed between the continuous sleep variables (sleep duration, sleep variability, and sleep efficiency) and food cravings, dietary quality, and BMI z-score. Independent samples t-tests were conducted between groups to compare continuous measures of sleep, food craving, and dietary quality between those meeting vs. not meeting sleep guidelines, between those who had a regular vs. late bedtime, and between those with and without obesity. A series of independent linear regression models were conducted between each sleep variable (sleep duration, meeting sleep guidelines, sleep variability, sleep efficiency, and late bedtime) and each dependent variable of food craving (subcomponent and total scores), dietary quality (HEI total score and energy intake), and BMI z-score. The association between craving and dietary quality (or BMI z-score) was assessed in independent linear models. Logistic regression was used to assess the relationship between sleep and food cravings with obesity. Linear and logistic regression models were adjusted for age, sex, race (white, African American, or other), in school or on holiday status, and MVPA (average minutes per day). These covariates were selected as each of them was significantly associated (*p* < 0.05) with at least two of the variables examined (i.e., sleep, food cravings, dietary quality, or BMI z-score).

#### Mediation Analysis, Multiple Comparison Adjustment, and Sensitivity Analysis

Mediation analysis was planned when a specific sleep variable was found to be associated with both food craving and a dietary quality or BMI z-score in independent models, and this same food craving was associated the same dietary quality or BMI z-score. Total mediation was achieved if the sleep variable was no longer significant in the same model as the craving score variable based on previous methods [32]. The Benjamini-Hochberg procedure applied to correct for multiple comparisons [33], specifically through the application of a false discovery rate of 25% to all results. After accounting for the false discovery rate, the revised *p*-value used in this analysis was 0.029.

Adolescents who completed at least one dietary recall were included in the initial analysis. A sensitivity analysis was completed with those who completed all three days of dietary recall. All analyses were performed using SAS 9.4.

## 3. Results

In total, 342 participants completed baseline measurements. Those with missing data were excluded from the analytic sample, including those who did not wear the accelerometer overnight or had fewer than 3 days of sufficient sleep data (*n* = 43), reported “never” for all craving responses (*n* = 5), missed individual craving questions (*n* = 5), did not record any dietary intake (*n* = 21), had no recorded anthropometry (*n* = 2), or had sufficient sleep data but did not have 4 days (including 1 weekend day) with at least 10 h of daily movement data (*n* = 10). Demographic characteristics of the 256 participants included in the analytic sample are provided in Table 1.

On average, the sample was balanced for sex (45.3% male), with 58.2% of participants identifying as white and 60.2% of participants reported being in school during the study. About half of the participants met the sleep duration guidelines for their age (51.5%) and most averaged a bedtime later than 10:00 pm (81.3%). The proportions of the sample with overweight and obesity were 12.3% and 35.5%, respectively. The average HEI total score was 48.1 ± 11.4, which is a relatively low score overall, as HEI total score ranges from 0–100. There were no differences in age, demographic characteristics, sleep measures, craving scores, dietary quality or BMI z-scores between the analytic sample and those not included due to missing data (*n* = 86) when compared using independent samples t-tests and chi-square analysis.

### 3.1. Sleep and Cravings

Correlations between sleep, food craving, dietary quality, and BMI z-scores are presented in Table 2, and results of the linear regression models predicting sleep are displayed in Table 3. Sleep duration, variability, and efficiency were not significantly correlated with each other (*p* > 0.05, data not shown). In unadjusted correlations, greater sleep efficiency was related to lower cravings for all indices (high fat, sweets, carbohydrate/starch, FCI-28, and FCI-33) except fast food and fruit/vegetable cravings, and with energy intake (*p* < 0.029 for all, Table 2). After adjustment for covariates, sleep efficiency was inversely associated with sweets (*β* = −10.68, *SE* = 4.50, *p* = 0.01) and FCI-28 (*β* = −8.35, *SE* = 3.41, *p* = 0.01). In other words, adolescents who spent a greater proportion of the total sleep period in sleep had less frequent cravings for sweets and foods evaluated except fruit/vegetable (FCI-28). There were no other significant associations between the sleep variables and cravings, including independent t-tests with sleep guidelines and late bedtime.

### 3.2. Sleep, Dietary Quality, and Obesity

Longer sleep duration was associated with a higher (better quality) HEI total score in both unadjusted correlations and adjusted models (*p* = 0.005 and *p* = 0.004, respectively). After adjustment for confounders, each additional hour of sleep was associated with a 2.04 (*SE* = 0.71) point higher HEI total score. Adolescents who met the sleep guideline had a higher HEI total score compared to those who did not by 3.69 points (*SE* = 1.45, *p* = 0.01) in adjusted models. Longer sleep duration was negatively associated with BMI-z score in both unadjusted (*p* = 0.005) and adjusted models (*β* = −0.16, *SE* = 0.07, *p* = 0.02). Sleep variability was associated with energy intake, as sleeping an additional hour on the weekend compared to the weekday was associated with 189 additional kilocalories of energy intake (*p* = 0.02). Having a late bed time was associated with a lower HEI total score (*β* = −4.20, *SE* = 1.80, *p* = 0.02) in unadjusted comparisons, but this difference was no longer significant after adjustment (*p* = 0.06). Sleep efficiency was not associated with dietary quality or obesity.

### 3.3. Cravings, Dietary Quality, and Obesity

Adjusted associations between food cravings, dietary quality, and obesity are presented in Table 4. Fast food cravings were negatively associated with HEI total score (*p* = 0.01) and fruit/vegetable cravings were positively associated with HEI total score in adjusted models (*p* = 0.003, Table 4). There were no other significant associations between craving scores and dietary quality or BMI z-scores.

### 3.4. Sensitivity Analysis

A sensitivity analysis was performed on those with complete dietary data (3 dietary recalls; *n* = 137). This sub-sample included significantly more males (63.7%, *p* = 0.01), more White adolescents (61.7%, *p* = 0.01) and reported higher energy intake (1914 ± 760, *p* = 0.001) compared to the remainder of the sample. The sub-sample did not differ on other demographic, sleep, craving, dietary quality or weight variables. The sensitivity analysis confirmed results of the main analysis, though four relationships became non-significant. In independent models, sleep duration (*β* = 0.98, *SE* = 0.94, *p* = 0.29), meeting the sleep duration guidelines (*β* = 1.82, *SE* = 1.88, *p* = 0.32), and fast food cravings (*β* = −1.83, *SE* = 1.04, *p* = 0.08) were no longer significantly associated with HEI total score. Sleep duration was no longer associated with BMI z-score (*p* = 0.55). Additionally, an association was found between sleep efficiency and high fat cravings (*β* = −11.56, *SE* = 4.95, *p* = 0.02), and total cravings (*β* = −9.63, *SE* = 3.94, *p* = 0.01). Further negative associations were found between sleep variability with sweet (*p* = 0.02) and fast food cravings (*p* = 0.01), but no longer associated with energy intake (*p* = 0.20). Having a late bedtime with less frequent fruit/vegetable cravings (*p* = 0.01). In sum, sleep duration was no longer significant, though relationships between other sleep parameters with food cravings and energy intake persisted. However, there was still no evidence of a mediation model between sleep, food cravings, dietary intake/quality, and obesity.

### 3.5. Mediation Model

No specific sleep parameter was associated with both cravings and dietary quality or BMI z-score. Therefore, there was no evidence for a consistent association between a specific sleep variable, craving, and dietary quality or between sleep, craving, and obesity, and thus the mediation models proposed a priori were not performed.

## 4. Discussion

In this cross-sectional study of adolescents, better sleep efficiency was associated with less food cravings for several food groups. Sleep duration, meeting the sleep duration guidelines, and fruit/vegetable cravings were positively associated with dietary quality, while fast food cravings were negatively associated with dietary quality. Similar to previous studies using self-report [6,19], longer sleep duration was associated with lower BMI z-scores. Because of a lack of consistent associations among variables, it was not possible to test a mediation model linking sleep, craving, and dietary quality or obesity. Sleep was related to cravings, dietary quality, and weight through differing mechanisms, reinforcing the need for adequate quality and quantity of sleep in adolescence.

Sleep efficiency was the only sleep metric related to cravings in this study. Interestingly, this is among the first studies to observe an association linking poor sleep efficiency with higher cravings, particularly in the adolescent age range. A prior study in 57 adults observed that higher daytime sleepiness was related to higher savory cravings and a higher wanting for high fat and sweet foods [13]. Sleepiness was not evaluated in the present study, but symptoms of sleepiness (foggy and sleepy) may be a result of poor sleep quality or inefficient sleep.

It is unclear how sleep efficiency might affect unhealthy food cravings. One potential mechanism may be that insufficient continuous sleep detrimentally affects brain development including the prefrontal cortex, which controls food cravings [34] and continues to develop throughout adolescence [35]. Poor sleep efficiency may influence the brain’s ability to recover during the sleep period and impair the brain’s ability to regulate. For example, a recent systematic review concluded that poor sleep quality in school-aged children and adolescents was associated with higher activity in brain regions that involved impulsivity and risk-taking, including the prefrontal cortex [36]. Therefore, the lack of continuous sleep periods may potentially lead to less recovery time for the prefrontal cortex. By contrast, sleep duration was not related to cravings in the present study; this may be because total sleep duration can mask intermittent periods of non-sleep. These mechanisms warrant further exploration in prospective trials.

Interestingly, while sleep efficiency was related to cravings, sleep efficiency was not related to dietary quality or BMI z-scores. A potential explanation is that the adolescents in the present cohort had a higher and less variable sleep efficiency (range of 0.91 to 0.99), namely due to the waist worn protocol, compared to a cross-sectional sample of 829 adolescents (0.84 median) [37]. However, the range of sleep efficiency was similar to other studies using the same validated algorithm in a cross-sectional cohort of 5873 children ages 9 to 11 years [38]. Considering the age of this population, there are other behaviors that influence sleep efficiency and dietary quality, such as screen-time habits [39]. In other cross-sectional studies of adolescents, greater than two screens in the bedroom was related to lower sleep efficiency [39], and a high amount of television viewing was associated with lower dietary quality [40]. Therefore, while sleep efficiency itself may be related to certain food cravings, other adolescent behaviors may play a larger role in the relationship between sleep efficiency and diet or obesity at this age. If there is a relationship between sleep efficiency and diet or obesity, longer-term studies in samples with poor sleep efficiency and assessment of screen-time may be required to detect associations.

Sleep duration was another important sleep variable examined in the present study. Sleep duration was related to dietary quality, aligning with previous evidence suggesting shorter sleep duration may be associated with higher energy intake in the evening [7,41] and breakfast skipping [7,9]. As cravings were not found as an intermediary between sleep duration and dietary quality, the timing and quality of diet and sleep may be the main mechanism at work. This relationship may be considered with the “hedonic” theory that regulates consumption and influences sleep, whereby those who sleep for shorter durations have more awake time to consume food. The relationship between sleep variability and energy intake may also relate to the hedonic theory, as those with shorter sleep on the weekdays (relative to weekends) may have additional time to consume food, compared to those with similar amounts throughout the week. As the relationship between sleep and dietary quality is complex, there is another mechanism to be considered. Another proposed mechanism, a “homeostatic theory,” postulates that shorter sleep leads to increased ghrelin and decreased leptin levels, thus increasing an appetite-stimulating hormone while decreasing an appetite-suppressing hormone [7]. This theory is mainly based on an experimental study of 12 healthy young men that found sleep restriction was associated with lower leptin and higher ghrelin [42]. Further, higher ghrelin was associated with higher total food cravings and specifically carbohydrate/starch cravings in another prospective study of 339 adults [17]. Further research is needed to examine both the hedonic and homeostatic theories in adolescents, and the role cravings may play.

The relationship between sleep and obesity is a topic of recent interest, and previous systematic reviews have found a relationship between sleep duration (or meeting sleep duration guidelines) and obesity in children [43,44]. Similar results were found in the current study with sleep duration being negatively associated with BMI z-score. However, meeting the sleep duration guideline *per se* was not associated with BMI z-score or obesity. One difference from prior studies is that the present study used an objective measure of sleep whereas others have used self-report, which may overestimate sleep [45] or may tap into the child’s or parent’s perceptions of sleep that are based on bedtime rather than actual time spent sleeping. Regardless, an adequate amount of sleep should be recommended for all adolescents due to its benefits on health and other behaviors, including memory and emotional regulation [43], and prospective trials are needed to examine the influence of sleep on obesity beyond cross-sectional associations.

To our knowledge, this is among the first studies to link subjective food cravings to diet quality in any population. Therefore, this study contributes new evidence that more frequent cravings for fruits and vegetables were associated with higher dietary quality in adolescents, while more frequent cravings of fast food were associated with a lower dietary quality. The dietary quality score for this sample was lower than observed in other adolescent samples ages 11 to 16 years [46], potentially because of the use of weekend days which may have lower dietary quality [47]. Another sample of young adolescents (ages 8 to 12 years) reported similar HEI total scores [48]. Interestingly, there was only one unhealthy food craving (fast food) related to poor dietary quality, and dietary quality has been the main mechanism proposed for the relationship between frequent cravings in persons with obesity [49]. Most experimental research in children to young adults has focused on general unhealthy cravings (such as donuts, pizza, cookies, salty and sweet foods) via this hypothesis [14,50], whereas the present study indicated a craving for specific healthy (fruit/vegetable) and unhealthy (fast food) food items was related to dietary quality. Dietary quality is lower between early childhood (ages 4 to 8 years) and older adolescence (ages 14 to 18 years) in cross-sectional studies [3]. During this time, fruit and vegetable consumption also declines, while sodium and refined grains consumption increases [3]. Coupled with present findings, additional longitudinal research is needed in this age group to better understand the links between specific food cravings and dietary quality, namely if these cravings change throughout adolescence and are related to dietary quality.

Taken together, this study did not find evidence of a single mediation model between sleep, cravings, and diet or obesity. A recent study in toddlers found evidence of parts of the mechanism, as different components of sleep (i.e., duration, bedtime delay, and quality) were related to separate eating behaviors, but there was no consistent association between sleep and obesity-related behaviors [51]. These authors suggested there may be differing eating behavior mechanisms and theories related to obesity development [51]. More specifically, the authors hypothesized that a lower amount of sleep may influence other neural and hormonal mechanisms, and poor sleep quality may influence emotional overeating. In the current study, there was no evidence of associations between sleep duration and cravings to support these neural or hormonal mechanisms. However, sleep efficiency, which is a marker of sleep quality, was associated with cravings, which highlights the importance of continuous sleep for adolescents. Sleep should be considered as a multicomponent behavior, both in terms of quality and quantity, as this behavior may relate to adequate food response and diet quality.

Strengths of the current study include the objective measure of sleep and use of a previously validated questionnaire to assess specific food cravings. Though the gold standard of sleep measurement is polysomnography, and future studies could incorporate this standard for sleep measurement. Another strength of this study is the use of a three-day food recall and the HEI. HEI is a widespread and validated index [31,52] that assesses dietary quality while adjusting for calorie intake. Accordingly, in this study all of the reported daily recalls were used, including weekend days and weekdays. Adolescents may differ in energy intake on weekend vs. weekdays [47], and only one day of dietary intake may not be representative of average consumption. However, the sensitivity analysis, which only included participants who had three dietary recalls, did not change the majority of results. The cross-sectional design and potential for reverse causality are important limitations in this study. Most of the dietary recalls were collected prior to the clinic visit, thus the craving measurement did occur after most days were tracked. Further, the FCI did not specifically query on when cravings occurred, so it is not possible to determine the temporality of cravings with sleep behavior. However, the cravings instrument assessed cravings over the prior month, which may represent additional intake, but would capture cravings during the period of dietary recall. Longitudinal evaluation with multiple time points may better explain the relationships of sleep, cravings, and diet, including daily fluctuations and examining cravings for both healthy and unhealthy foods.

These findings suggest additional research is needed to fully understand the relationship between sleep, cravings, and diet as well as sleep, cravings, and obesity. It is possible a physiological, either hedonic or homeostatic theory, could explain the relationship between sleep duration and food cravings through action on the prefrontal cortex of the brain [35]. Accordingly, future research should include the use of sleep, specific food cravings, and hormone measurements to investigate any “homeostatic” theory that is occurring. Diet timing is also an important aspect to consider and was not explored in this study. Therefore, incorporation of diet timing, polysomnography, hormone measurement, and cravings over a shorter time span (such as a day or week) may best capture the entire mechanism. Considering the adolescent age range, screen-time may negatively contribute to sleep efficiency, including text messages and media use after bedtime [53]. Media use may also be related to dietary quality [54] and energy intake [55], thus this additional behavior may need to be explored in the context of cravings. The family environment is also important at this age and is related to dietary quality. As the family environment may influence the frequency and availability of food [46], additional investigation into the family influence on this mechanism is needed.

## 5. Conclusions

Overall, this study of adolescents found that poor sleep efficiency was related to unhealthy food cravings, and shorter sleep duration was related to lower dietary quality and higher body weight. Notably, cravings for fast food and fruits/vegetables were found to be associated with dietary quality, but together this study did not find a specific joint relationship between sleep, cravings, diet, and obesity. Sleep and diet remain important during the adolescent time period, and additional longitudinal research is needed to fully understand the relationship of the quantity and quality of sleep with cravings, dietary intake/quality, and obesity.

## Figures and Tables

**Table 1:** Descriptive Characteristics of Adolescent Participants (*n* = 256).

		Mean	SD	%
*Demographics*				
Age (years)		12.4	1.9	
Male				45.3
Race	White			58.2
	African American			36.3
	Other			5.5
Household Income	Less than $29,000			8.5
	$30,000–70,000			27.3
	$71,000–140,000			34.0
	$140,000 or more			23.9
	Missing/Refused			6.3
In School (vs. on school holiday)				60.2
*Sleep Variables*				
Sleep Duration, hours/day		8.7	1.0	
Met Sleep guidelines				50.4
Sleep Variability, hours		0.2	0.5	
Sleep Efficiency		0.96	0.01	
Late Bedtime				81.3
*Craving and Diet Quality*				
High Fat		2.4	0.9	
Sweets		2.9	0.9	
Carbohydrate/Starch		2.6	0.8	
Fast Food		3.0	0.9	
FCI - 28		2.7	0.7	
Fruit/Vegetable		2.5	0.8	
FCI-33		2.6	0.6	
HEI Total Score		48.1	11.4	
Energy Intake (kilocalories)		1756	748	
*Body Weight Classification*				
BMI z-score		0.8	1.2	
BMI Classification	Underweight			3.2
	Normal Weight			49.0
	Overweight			12.3
	Obese			35.5

**Table 2:** Pearson correlations between Sleep, Food Cravings, and Dietary Quality (*n* = 256).

	Sleep Duration	Sleep Variability	Sleep Efficiency	HEI Total Score	Energy Intake	BMI Z-Score
*Food Cravings*						
High Fat	−0.03	0.01	−0.14 *	−0.01	0.11	0.01
Sweets	0.00	0.01	−0.15 *	−0.02	0.11	−0.12
Carb/Starch	0.00	0.03	−0.13 *	−0.02	0.03	0.04
Fast Food	−0.08	−0.01	−0.13	−0.15	0.04	0.07
FCI-28	−0.02	0.02	−0.17 *	−0.05	0.09	−0.01
Fruit/Vegetable	0.00	−0.06	0.02	0.018 *	−0.01	0.04
FCI-33	−0.02	0.00	−0.14 *	−0.01	0.08	0.00
*Dietary Intake/Quality*						
HEI total score	0.17 *	0.03	0.02		−0.02	−0.05
Energy Intake	0.00	0.08	−0.15 *	−0.02		−0.06
*Body Weight Classification*						
BMI z-score	−0.21 *	0.00	−0.02	−0.05	−0.06	

FCI: Food Craving Inventory; * significant correlation between variables (*p* < 0.029).

**Table 3:** Beta Coefficients for the Associations of Sleep with Food Cravings, Dietary Quality, and Obesity in Adolescents (*n* = 256)^.

***Sleep and Cravings***												
	**High Fat**			**Sweets**			**Carbohydrates/Starch**			**Fast Food**		
	**Beta**	***SE***	***p*-Value**	**Beta**	***SE***	***p*-Value**	**Beta**	***SE***	***p*-Value**	**Beta**	***SE***	***p*-Value**
Sleep Duration	−0.02	0.04	0.66	0.009	0.05	0.86	0.03	0.04	0.48	−0.02	0.05	0.61
Met Sleep Guidelines	−0.03	0.09	0.70	−0.08	0.11	0.48	−0.05	0.09	0.59	−0.18	0.11	0.11
Sleep Variability	−0.03	0.08	0.73	−0.008	0.10	0.93	0.01	0.09	0.90	−0.02	0.10	0.78
Sleep Efficiency	−7.01	3.77	0.06	−10.68 *	4.50	0.01	−6.89	3.85	0.07	−9.39	4.44	0.03
Late Bedtime	0.08	0.12	0.50	0.11	0.14	0.45	0.10	0.12	0.40	0.20	0.14	0.16
	**FCI-28**			**Fruit/Vegetable**			**FCI-33**					
	**Beta**	***SE***	***p*-Value**	**Beta**	***SE***	***p*-Value**	**Beta**	***SE***	***p*-Value**			
Sleep Duration	0.002	0.04	0.95	0.03	0.05	0.52	0.007	0.04	0.85			
Met Sleep Guidelines	−0.07	0.01	0.39	−0.008	0.10	0.93	0.06	0.8	0.45			
Sleep Variability	−0.01	0.08	0.88	−0.09	0.10	0.33	−0.02	0.07	0.74			
Sleep Efficiency	−8.35 *	3.41	0.01	1.22	4.26	0.77	−6.90	3.21	0.03			
Late Bedtime	−0.11	0.11	0.31	0.10	0.13	0.47	−0.11	0.10	0.28			
***Sleep, Diet Quality and Obesity***												
	**HEI Total Score**			**Energy Intake**			**BMI z-Score**			**Obesity ^#^**		
	**Beta**	***SE***	***p*-Value**	**Beta**	***SE***	***p*-Value**	**Beta**	***SE***	***p*-Value**	**Beta**	***SE***	***p*-Value**
Sleep Duration	2.04 *	0.71	0.004	−31.02	46.2	0.50	−0.16 *	0.07	0.02	−0.26	0.14	0.07
Met Sleep Guidelines	3.69 *	1.45	0.01	−21.02	94.2	0.82	0.18	0.15	0.22	−0.15	0.14	0.85
Sleep Variability	−0.33	0.35	0.80	189.4	85.6	0.02 *	−0.01	0.13	0.93	−0.12	0.26	0.63
Sleep Efficiency	−0.78	57.4	0.98	6536	3641	0.07	−9.69	5.87	0.10	−14.45	11.4	0.20
Late Bedtime	−3.43	1.86	0.06	26.44	119.7	0.82	0.009	0.19	0.97	0.01	0.18	0.92

FCI: Food Craving Inventory; ^ Assessed using generalized linear regression. Adjusted for age, sex, race, in-school/out of school status, and MVPA (minutes), ^#^ Assessed using logistic regression, with adjustment for age, sex, race, in-school/out of school status and MVPA (minutes). * *p* < 0.029.

**Table 4:** Beta Coefficients for the Associations of Food Cravings with Dietary Quality and Obesity in Adolescents (*n* = 256)^.

	HEI Total Score			Energy Intake			BMI Z-Score			Obesity ^#^		
	Beta	*SE*	*p*-Value	Beta	*SE*	*p*-Value	Beta	*SE*	*p*-Value	Beta	*SE*	*p*-Value
High Fat	−0.37	0.95	0.69	93.40	60.9	0.12	0.05	0.09	0.57	−0.04	0.18	0.80
Sweets	−0.33	0.80	0.67	81.0	50.8	0.11	−0.15	0.08	0.06	0.16	0.15	0.28
Carbohydrates/Starch	−0.29	0.93	0.75	20.5	59.9	0.73	0.07	0.09	0.45	−0.05	0.18	0.74
Fast Food	−1.92 *	0.80	0.01	34.7	51.8	0.50	0.04	0.08	0.63	0.009	0.15	0.95
FCI-28	−0.86	1.05	0.41	88.28	67.2	0.18	−0.02	0.10	0.82	0.04	0.20	0.81
Fruit/Vegetable	2.49 *	0.84	0.003	3.16	54.55	0.95	0.02	0.08	0.74	−0.05	0.16	0.73
FCI-33	−0.18	1.12	0.87	85.8	71.5	0.23	−0.01	0.11	0.89	0.03	0.21	0.88

^ Assessed using generalized linear regression. Adjusted for age, sex, race, in-school/out of school status, and MVPA (minutes), ^#^ Assessed using logistic regression, with adjustment for age, sex, race, in-school/out of school status and MVPA (minutes). * *p* < 0.029.
